# Perivascular network segmentations derived from high-field MRI and their implications for perivascular and parenchymal mass transport in the rat brain

**DOI:** 10.1038/s41598-023-34850-0

**Published:** 2023-06-06

**Authors:** Julian A. Rey, Uzair M. Farid, Christopher M. Najjoum, Alec Brown, Kulam Najmudeen Magdoom, Thomas H. Mareci, Malisa Sarntinoranont

**Affiliations:** 1 Department of Mechanical and Aerospace Engineering, University of Florida, PO BOX 116250, Gainesville, FL 32611 USA; 2 Department of Biochemistry and Molecular Biology, University of Florida, Gainesville, FL USA

**Keywords:** Permeation and transport, Image processing, Biophysical models, Biomedical engineering, Neuroscience

## Abstract

A custom segmentation workflow was applied to ex vivo high-field MR images of rat brains acquired following in vivo intraventricular contrast agent infusion to generate maps of the perivascular spaces (PVS). The resulting perivascular network segmentations enabled analysis of perivascular connections to the ventricles, parenchymal solute clearance, and dispersive solute transport within PVS. Numerous perivascular connections between the brain surface and the ventricles suggest the ventricles integrate into a PVS-mediated clearance system and raise the possibility of cerebrospinal fluid (CSF) return from the subarachnoid space to the ventricles via PVS. Assuming rapid solute exchange between the PVS and CSF spaces primarily by advection, the extensive perivascular network decreased the mean clearance distance from parenchyma to the nearest CSF compartment resulting in an over 21-fold reduction in the estimated diffusive clearance time scale, irrespective of solute diffusivity. This corresponds to an estimated diffusive clearance time scale under 10 min for amyloid-beta which suggests that the widespread distribution of PVS may render diffusion an effective parenchymal clearance mechanism. Additional analysis of oscillatory solute dispersion within PVS indicates that advection rather than dispersion is likely the primary transport mechanism for dissolved compounds greater than 66 kDa in the long (> 2 mm) perivascular segments identified here, although dispersion may be significant for smaller compounds in shorter perivascular segments.

## Introduction

Blood vessels in the brain are surrounded by slender perivascular spaces (PVS) that enable fluid exchange between interstitial fluid and cerebrospinal fluid (CSF) compartments^1^. These structures have garnered much attention recently because of the role they may play in a brain-wide clearance mechanism for toxic metabolic waste such as amyloid-beta (Aβ), the protein that accumulates in Alzheimer’s disease^2^. While rapid imaging tracer uptake from the CSF^3,4^ and clearance from parenchyma^5^ have been observed, there is uncertainty^1^ regarding the mechanism and direction of transport^6–8^, the anatomy of arterial, capillary, and venous perivascular transport routes^5^, and the effect of aquaporin water channels^2^ and sleep^9^ on transport. Nonetheless, PVS-mediated transport in the brain may have significant implications not only for neurodegenerative diseases, but also for drug delivery to brain tissue^10^ and the migration of brain cancer^11,12^ and immune cells^13^.

While a number of studies have demonstrated uptake of imaging tracers into PVS near the brain surface^3,4,14^, few have examined deeper PVS and their connections to CSF in the cerebral ventricles and cisterns^15,16^. Histological sections following tracer uptake suggest an intricate and extensive network of PVS throughout the brain^17^, but the resolution of whole-brain imaging in vivo has limited analysis of the intact perivascular network to only the largest vessels^17–19^. There is a need for a whole-brain 3D map of major perivascular structures to analyze perivascular network properties relevant to clearance such as connections to internal CSF spaces, the distribution of PVS in parenchyma, and perivascular segment length. A map of PVS would enable potential perivascular and parenchymal transport mechanisms such as diffusion, dispersion, and advection to be evaluated via multi-scale mechanical modeling. This is required to better understand waste clearance and to accurately plan a variety of drug delivery techniques including intravenous, intrathecal, and convection-enhanced delivery to parenchyma.

Although a segmentation of the intact perivascular network in rats has not been published to the authors’ knowledge, several semi-automatic strategies have been developed to segment human PVS in clinical MR images^20–25^. In many of these strategies, the “tubeness” or “vesselness” of the image is determined by applying Frangi filters^26^ that are based on the spatial curvature of the image intensity. By applying a threshold to these tubeness images, a segmentation of PVS is produced and incorporated into a larger segmentation methodology often relying on deep learning techniques^20,21^. Despite its prevalence in human perivascular segmentation, tubeness thresholding has not been previously applied to segment PVS in rats or mice mainly because the resolution of in vivo MR images acquired during cerebrospinal contrast agent administration is not sufficiently high to resolve most PVS containing the contrast agent.

Magdoom, et al.^16^ present a set of ex vivo rat brain images with 40 μm isotropic voxels acquired at 17.6 T following intraventricular contrast agent infusion, revealing numerous connections between the perivascular network and the cerebral ventricles. In the present study, these images were newly processed to produce clearer visualizations of the perivascular network that facilitate the identification of major blood vessels surrounded by patent PVS. A custom segmentation workflow relying on tubeness was developed to create high-resolution 3D maps of the PVS containing contrast agent following intraventricular infusion. The resulting maps were used to isolate those perivascular segments in the vicinity of the ventricles and cisterns which may serve as clearance routes from parenchyma to deep CSF spaces. The extent to which the PVS facilitate parenchymal waste clearance by reducing the transport distance to the nearest CSF sink was quantified. An analytical transport model was also developed to evaluate dispersion caused by oscillatory perivascular flow as a potential solute transport mechanism in the longest PVS segments measured.

## Results

**Figure 1:**
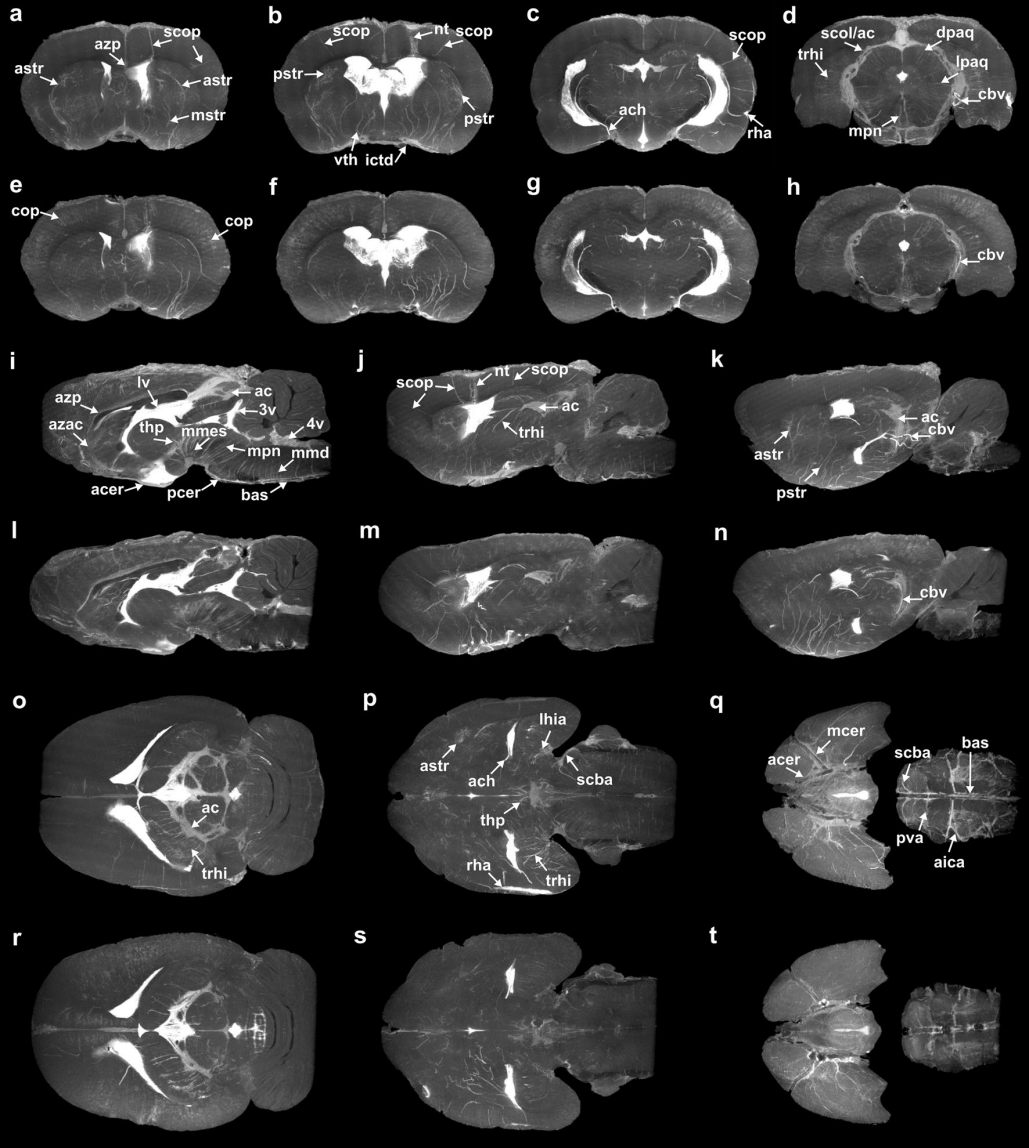
Partial maximum intensity projections (pMIPs). Each panel is a projection across 30 slices parallel to the coronal (**a–h**), sagittal (**i–n**), and transverse (**o–t**) planes for Rat 3 (**a–d**, **i–k**, and **o–q**) and Rat 4 (**e–h**, **l-n**, and **r–t**). Proceeding alphabetically, each coronal panel is posterior to the preceding panel, each sagittal panel is lateral to the preceding panel, and each transverse panel is ventral to the preceding panel for each animal. Notable vessels are labeled (see Supplementary Table S1).

Extensive perivascular uptake of Gd-DTPA-albumin throughout the brain was observed following intraventricular infusion. Long perivascular segments, some over 3 mm in length, extend from the surface of the brain to the deep tissues surrounding the ventricular system (Fig. 1). The PVS are visible as bright, slender streaks in the coronal (Fig. 1a–h, Supplementary Video S1 and S2), sagittal (Fig. 1i–n, Supplementary Video S3 and S4), and transverse (Fig. 1o–t, Supplementary Video S5 and S6) planes. PVS surrounding subcortical penetrating arteries (scop) traverse the cortex to the subcortical white matter and periventricular tissue thus providing potential pathways for tracer between the dorsal subarachnoid space and the lateral ventricles (Fig. 1a–c, f, j, m). Perivascular uptake along smaller cortical vessels (cop) likely occurred given the dense array of bright, radial streaks (Fig. 1e–h), though individual vessels are not easily distinguishable.

Perivascular uptake was evident along major ventral surface vessels including the anterior cerebral artery (acer), middle cerebral artery (mcer), posterior cerebral artery (pcer), internal carotids (ictd), and the basilar artery (bas) (Fig. 1b, f, i, q, t). Branches from these main vessels extending dorsally into the caudate and midbrain are surrounded by the brightest perivascular segments in the images and include the anterior (astr), medial (mstr), and posterior (pstr) striate arteries, ventral thalamic arteries (vth), and anterior choroidal arteries (ach) (Fig. 1a–c, e–g, j–k, m–n). A number of these PVS appear continuous with the ventricles (Fig. 1b, c, f–g) although the resolution is not sufficient to determine whether they are anatomically continuous with the ventricular CSF or they reside in periventricular tissue through which solutes could feasibly diffuse or flow to the ventricles. PVS surround numerous dorsally penetrating vessels in the midbrain and brain stem, especially near the longitudinal fissure, including the thalamo-perforating arteries (thp), median mesencephalic arteries (mmes), median pontine arteries (mpn), and the median medullary arteries (mmd) (Fig. 1i, l). The latter of these extend towards the fourth ventricle dorsal to the brainstem. PVS are also arranged radially about the aqueduct surrounding the lateral (lpaq) and dorsal (dpaq) periaqueductal arteries as well as the median pontine arteries (mpn) (Fig. 1d, h). In addition to strong contrast enhancement in the ventricles and PVS, weaker enhancement was present in the quadrigeminal and ambient cisterns (ac) (Fig. 1d, h, i–n, o, r). Contrast agent uptake in these cisterns is notable because they are continuous with external CSF spaces and are lined with numerous blood vessels^27^. The perivascular spaces surrounding some cisternal blood vessels (cbv) are evident in Fig. 1d, h, k, n.

**Figure 2:**
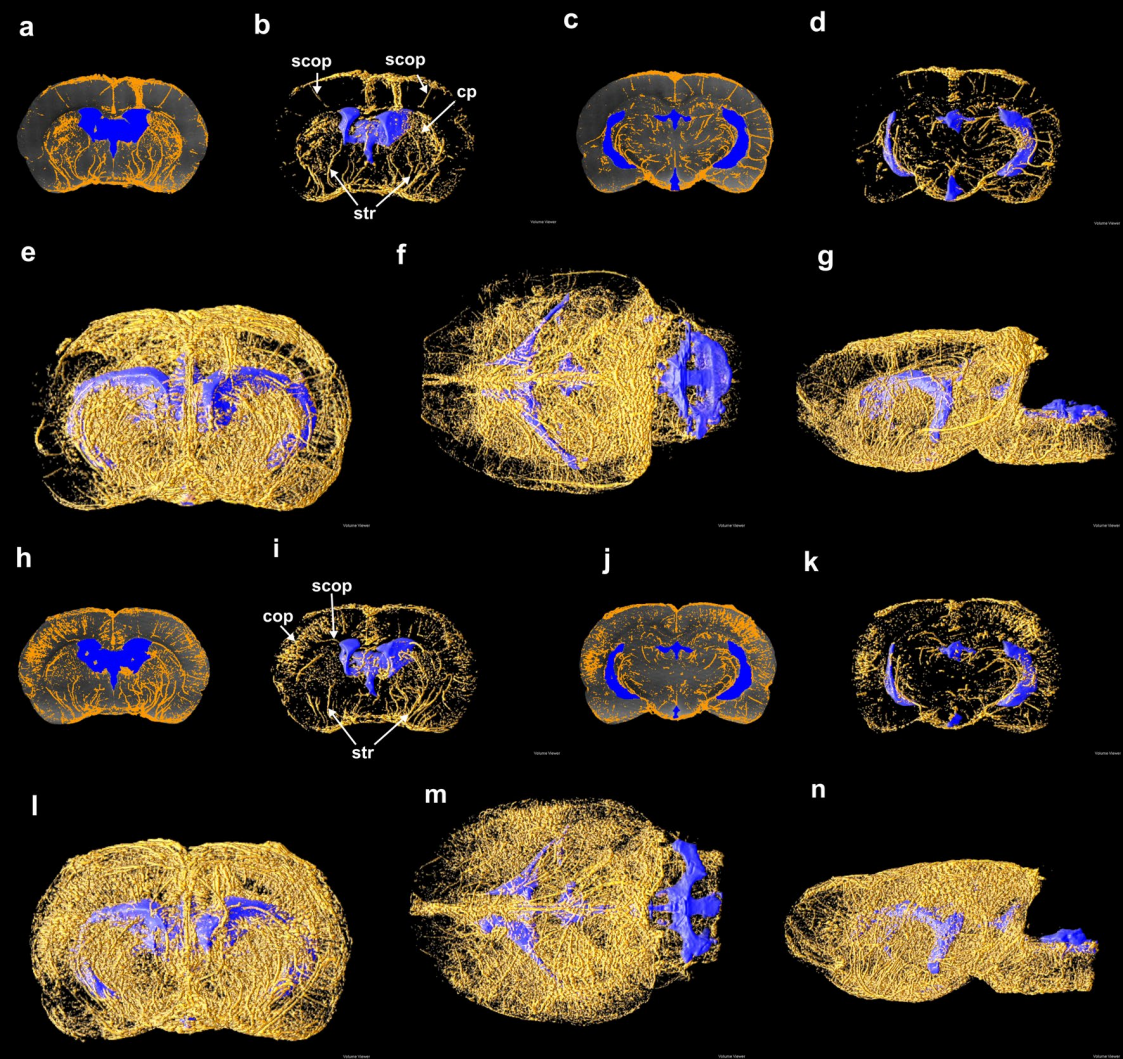
Perivascular and ventricle segmentations. The PVS (gold) and the ventricles (blue) segmented in two sets of 30 contiguous coronal slices are overlayed on the corresponding pMIPs for Rat 3 (**a, c**) and Rat 4 (**h, j**). These pMIPs are shown with overlayed segmentations for comparison with Fig. 1b, c, f, g, respectively. These 30-slice segmentations are presented alongside their 3D renderings for Rat 3 (**b, d**) and Rat 4 (**i, k**). All PVS (gold) and the ventricles (blue) are rendered in 3D for Rat 3 (**e–g**) and Rat 4 (**l–n**). Panels (**e**) and (**l**) are a rostral view of the coronal plane, (**f, m**) are a dorsal view of the transverse plane, and (**g**) and (**n**) are a left view of the sagittal plane. Notable vessels are labeled (see Supplementary Table S1).

**Figure 3:**
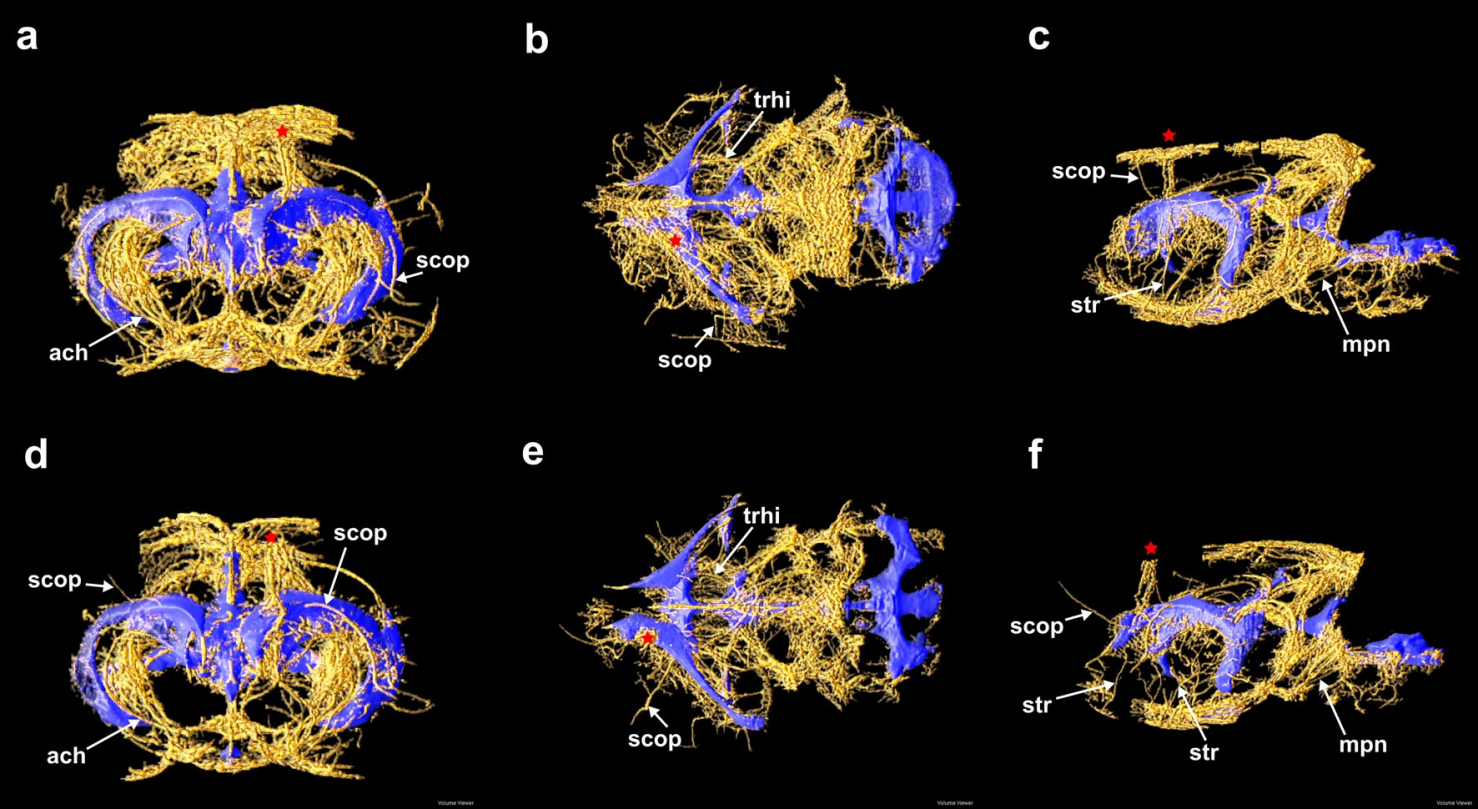
Connected perivascular spaces occupying periventricular regions. The portions of the perivascular network containing segments that traverse a three-voxel margin around the ventricles are 3D-rendered for Rat 3 (**a–c**) and Rat 4 (**d–f**). The left column is a rostral view of the coronal plane, the central column is a dorsal view of the transverse plane, and the right column is a left view of the sagittal plane. The needle track in each rat is labeled with a red star. Notable vessels are labeled (see Supplementary Table S1).

Image segmentation revealed a dense network of PVS throughout the brain (Fig. 2, Supplementary Fig. S1) and numerous PVS that extend from near the ventricles to the brain surface and cisterns (Fig. 3, labeled vessels). The segmented PVS in sets of 30 coronal slices (Fig. 2a, c, h, j) include the vast majority of the PVS visible in the pMIPs for the same 30 slices (Fig. 1b, c, f, g). The 3D structure of the perivascular segments (Fig. 2b, d, i, k) depicts both long segments (striate arteries and subcortical penetrating arteries) and shorter segments within the cortex and caudate putamen. These shorter segments appear to be both smaller perivascular spaces near the limit of the imaging resolution and vessels oriented perpendicular to the coronal plane in the caudate putamen. While the segmented PVS in the cortex, brainstem, and surrounding the aqueduct were generally less than 1 mm in length, several perivascular segments originating from surface vessels and terminating near the ventricles were over 2.5 mm in length. The solid renderings indicate that no parenchymal region is conspicuously devoid of PVS, although inter-animal variability in cortical uptake is apparent (Fig. 2e–g, l–m, Supplementary Fig. S1). The perivascular uptake of contrast agent in the cortex, striatum, periaqueductal tissue, and the brain stem was quantified by calculating the percent of voxels segmented as PVS within volumes of interest (VOI) in each anatomical region (Table 1). The highest average percent volume (2.86 ± 0.934) was observed in the striatum while the remaining regions had average percent volumes between 1.02 (periaqueductal tissue) and 1.89 (cortex). The percent segmented perivascular volume was 25% greater in the cortex of Rat 4 than in Rat 3 which is consistent with the 3D renderings in Fig. 2. The MR images for Rat 1 revealed that in this animal contrast agent was partially infused into the periventricular tissue. This likely explains the higher percent segmented perivascular volume in the striatum and lower percent segmented perivascular volume in the periaqueduct and brain stem, far from the infusion site.

**Table 1:** Percent volume of perivascular voxels and average minimum clearance distance (MCD) in select brain regions.

	Cortex		Striatum		Periaqueduct		Brain Stem	
	PVS percent volume	MCD (mm)	PVS percent volume	MCD (mm)	PVS percent volume	MCD (mm)	PVS percent volume	MCD (mm)
Rat 1*	1.28	0.266	4.37	0.149	0.349	0.337	0.609	0.313
Rat 2	1.55	0.209	3.03	0.143	0.975	0.222	1.61	0.195
Rat 3	1.85	0.297	2.51	0.154	1.34	0.180	1.63	0.195
Rat 4	2.32	0.165	2.52	0.151	1.34	0.209	1.29	0.196
Rat 5	2.45	0.167	1.88	0.154	1.08	0.197	1.71	0.175
Mean ± SD	1.89 ± 0.498	0.221 ± 0.0590	2.86 ± 0.934	0.150 ± 0.00438	1.02 ± 0.407	0.229 ± 0.0624	1.37 ± 0.456	0.215 ± 0.0556

The volumes of interest for each anatomical region span 30 coronal slices (Fig. 8). *In Rat 1, the contrast agent was partially infused into the periventricular parenchyma.

Isolating the portion of the perivascular network extending outward from within a narrow region at most three voxels from the ventricular surface revealed connections between the lateral ventricles and major ventral surface vessels via the anterior choroidal arteries and the striate arteries (Fig. 3a, c, d, f). A margin of three voxels was selected because this region is narrow enough to enable relatively rapid transport of perivascular tracers into the ventricles. Perivascular connections from the lateral ventricles to the dorsal surface via subcortical penetrating arteries (Fig. 3) are also apparent. The PVS surrounding the transverse hippocampal arteries visible in the transverse pMIPs (Fig. 1o, r) appear to connect the caudal boundary of the lateral ventricles to the ambient cistern (Fig. 3b, e). The fourth ventricle is near PVS that traverse the brain stem and extend rostrally (Fig. 3, f).

**Figure 4:**
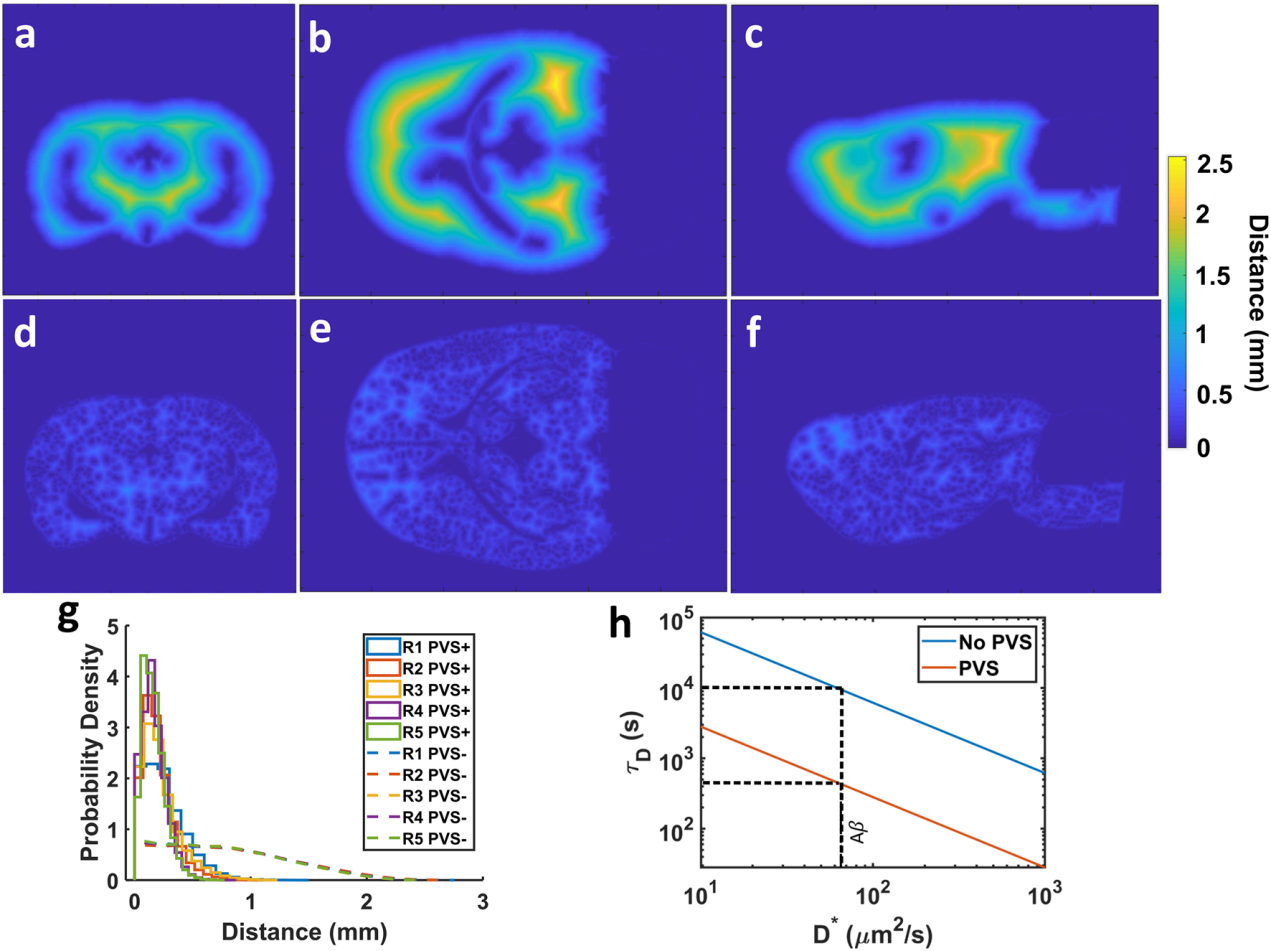
Minimum distance between parenchyma and CSF spaces. The distances between parenchyma voxels and the nearest CSF space without PVS (**a–c**) and including PVS (**d–f**) for Rat 4 are presented as planar color images. The left column is a rostral view of the coronal plane, the central column is a dorsal view of the transverse plane, and the right column is a left view of the sagittal plane. (**g**) Distributions of minimum distance between parenchyma voxels and the CSF spaces with and without PVS. (**h**) Diffusive transport time scales for a range of solute effective diffusivities with and without PVS.

The minimum distance between each parenchyma voxel and the nearest CSF space was computed both considering and not considering the PVS to be a CSF space (Fig. 4). In Rat 4, an animal exhibiting extensive perivascular tracer uptake, the parenchyma was on average 0.784 ± 0.516 mm away from the nearest CSF space in the absence of PVS, with some parenchymal regions located over 2 mm away from the nearest CSF space (Fig. 4g). These regions consisted of portions of the cortex and midbrain (Fig. 4a–c). When PVS were considered a CSF compartment, the mean minimum distance between parenchyma and the CSF was reduced to 0.169 ± 0.100 mm and all parenchyma voxels were within approximately 0.9 mm of a CSF space (Fig. 4g). This reduction in minimum distance represents an over 21-fold reduction in the time scale for diffusive clearance (see Eq. (2a)) from the parenchyma (Fig. 4h). The lowest average minimum clearance distance (MCD) across all animals was observed in the striatum VOI (0.150 ± 0.00438 mm) while the cortex, periaqueductal tissue, and brain stem VOI had values between 0.215 mm (brain stem) and 0.229 mm (periaqueduct) (Table 1). The maximum standard deviation of the MCD across animals was 62.4 μm in the periaqueductal tissue VOI. Supposing an interstitial flow were present in the parenchyma as posited by the glymphatic theory, the presence of PVS was estimated to reduce nearly five-fold the advective clearance time scale (see Eq. (2b)) from parenchyma.

**Figure 5:**
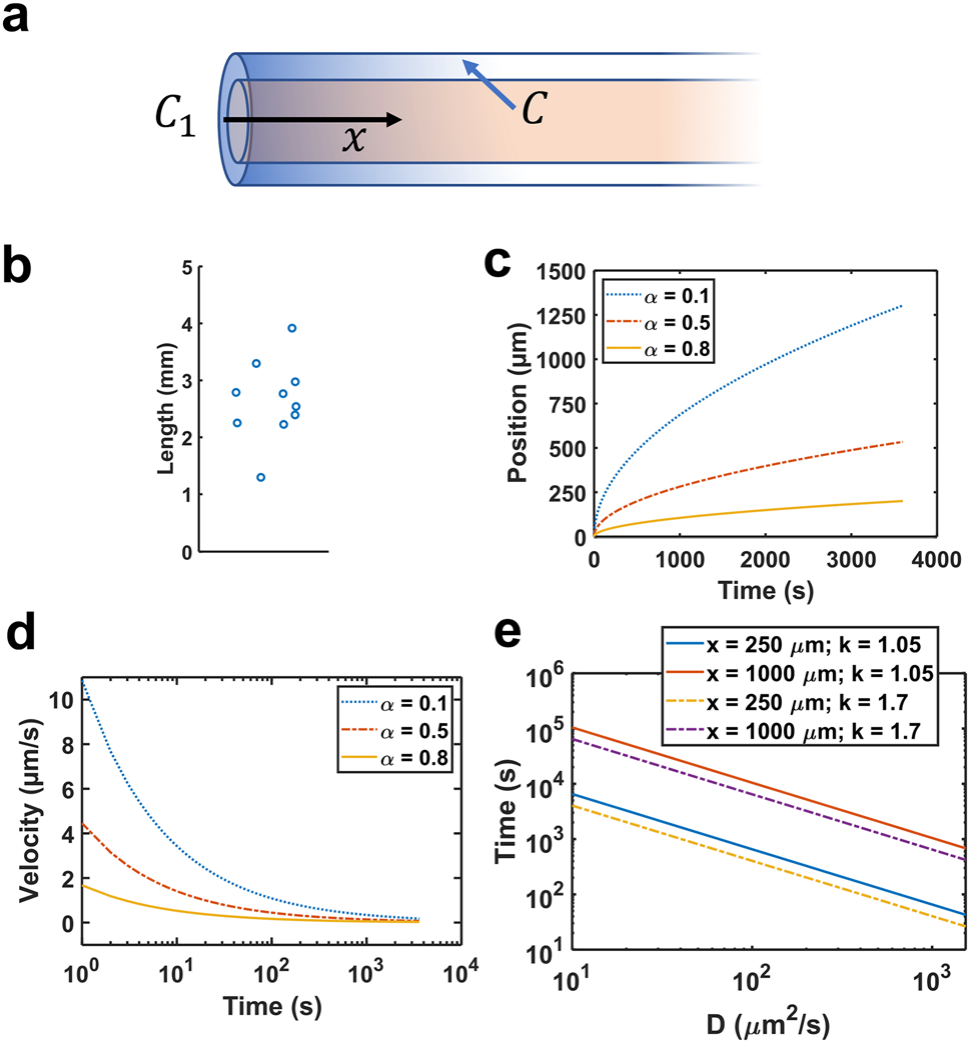
Oscillatory solute dispersion in PVS. (**a**) Dispersion model geometry consisting of a well-mixed CSF region with steady concentration $C_{1}$ and an adjacent semi-infinite straight perivascular segment initially at concentration $C$ = 0. (**b**) Measured lengths for a sample (n = 10) of the longest visually identifiable perivascular segments in Rat 5. The data points have been horizontally scattered to improve visibility. (**c**) Position and (**d**) velocity of advancing tracer fronts with concentrations $\alpha C_{1}$ over time (*k* = 1.05). (**e**) Time to traverse 250 μm and 1000 μm for a range of solute molecular diffusivities. Solid and dashed lines denote a 5%^7^ (*k* = 1.05) and 70%^6^ (*k* = 1.7) increase in the diffusion coefficient (dispersive enhancement) caused by oscillatory flow.

A transient transport analysis in a one-dimensional semi-infinite domain (Fig. 5a) was performed to ascertain whether oscillatory solute dispersion can explain the perivascular uptake observed in our intraventricular infusion experiment and following infusion into the cisterna magna as previously reported^2,14,19^. Assuming the CSF spaces were well-mixed and attained a high concentration shortly after the start of infusion, oscillatory perivascular flow resulted in an evolving perivascular concentration gradient with low-concentration leading regions traveling faster than trailing regions of higher concentration. These regions, or advancing solute fronts, are defined by their characteristic fraction $\alpha$ of the CSF concentration. For albumin, the $\alpha$ = 0.1 front traveled about 1 mm in 40 min whereas the $\alpha$ = 0.8 front traveled about 150 μm in the same amount of time (Fig. 5c). By comparison, the brains with contrast agent contained numerous segments well over 2 mm long, some up to 4 mm (Fig. 5b). The front velocity was maximum at $t$ = 0 because a step change in concentration was present at the boundary with the CSF (the concentration gradient was greatest here) and was just over 10 µm/s for $\alpha$ = 0.1. Within a minute, the velocity for all fronts with $\alpha$ > 0.1 dropped below 2 μm/s (Fig. 5d). The distance traveled due to oscillatory dispersion varies with the square root of the diffusion coefficient, meaning a four-fold change in this coefficient results in a two-fold change in the distance traveled. The times required for the $\alpha$ = 0.5 front to traverse perivascular lengths of 250 μm and 1000 μm for a physiologically relevant range of molecular diffusivities and two literature values of dispersive enhancement, $k$, are shown in Fig. 5e. The albumin solute front delayed between 8.11 min ($k$ = 1.7)^6^ and 13.14 min ($k$ = 1.05)^7^ to traverse 250 μm, but much longer (2.16 h at $k$ = 1.7) to traverse 1000 μm, consistent with the position vs. time plot ($k$ = 1.05) in Fig. 5c. The solute front for Aβ monomer ($D$ = 180 μm^2^/s)^28^, being smaller than albumin, traversed both distances more quickly, but still delayed 1.00 h ($k$ = 1.7) and 1.62 h ($k$ = 1.05) to travel 1000 μm. The solute front for sodium ions traversed 1000 μm in 11.36 min ($k$ = 1.05).

## Discussion

### Perivascular connections to the ventricles

A rapidly growing body of literature suggests the PVS communicate with the CSF spaces to form a brain-wide fluid exchange and waste clearance system^1,29^. Several pre-clinical studies have focused on dynamic tracer uptake into cortical PVS from the subarachnoid space using either in vivo fluorescence microscopy or post-mortem histology^2,4,5^. While cranial window microscopy enables high-resolution, real-time tracking of perivascular transport, a fixed field of view and shallow penetration depth limit analysis to a small, superficial portion of the perivascular network. As a result, the extent and connectivity of the brain-wide perivascular network and its functional relationship with the cerebral ventricles and cisterns are poorly understood. Bedussi, et al.^15^ observed PVS-mediated clearance to the ventricular system following infusion into the striatum, suggesting the ventricles function as a clearance sink for deep parenchymal tissue. Strong tracer signal in periventricular and cisternal tissues, as well as in a dense network of perivascular structures near the ventricles has also been observed following infusion into the cisterna magna^17^. These results imply that tracer arrives at the ventricles via deep parenchymal PVS since upstream intra-ventricular transport against ventricular flow seems less plausible. Indeed, dynamic contrast-enhanced MRI (DCE-MRI) data acquired during intracisternal infusion show that contrast agent moves through superficial perivascular spaces throughout the brain prior to arriving at the cerebral aqueduct indicating that minimal tracer moves upstream through the ventricular system during the infusion^18^.

In this study, we address the need for a high-resolution 3D visualization of the rat perivascular network and its connections to internal CSF spaces using high-field ex vivo MR images. While in vivo perivascular uptake^17–19^ of contrast agent following infusion into the cisterna magna has been observed with DCE-MRI, the imaging resolution in these studies limited analysis to large surface vessels. Maximum intensity projections through sub-volumes of the high-resolution (40 μm isotropic voxels) ex vivo MR images from Magdoom, et al.^16^ revealed numerous perivascular segments appearing to provide a direct pathway between the exterior surfaces of the brain and the deep tissues surrounding the cerebral ventricles (Fig. 1a–c, e–g). A number of these segments were between 2 and 4 mm in length (Fig. 5b). On the dorsal surface these include the subcortical penetrating arteries (scop), some of which, after traversing the cortex, run parallel to white matter tracks. Perivascular branches from large ventral surface vessels including the anterior choroidal arteries (ach), the striate arteries (astr, mstr, pstr), and the median medullary arteries (mmd) appeared to pass closely to or terminate near the ventricular surfaces, although the resolution is not sufficient to determine whether they are anatomically continuous with the ventricular CSF or they reside in periventricular tissue through which solutes could feasibly diffuse or flow to the ventricles. Numerous perivascular connections between the ambient cistern and the ventricles along trans-hippocampal arteries (trhi) and periaqueductal arteries (lpaq, dpaq) were evident. The clearance function of the trhi PVS may be especially relevant to Alzheimer’s disease given that plaque formation and atrophy in the hippocampus is a feature of the disease^30^.

The geometries of the PVS penetrating the brain from major ventral and dorsal surface vessels are consistent with published rat and mouse cerebrovascular atlases. Extensive images of the rat vasculature presented in Scremin^31^ were referenced when identifying the blood vessel groups that may provide perivascular solute pathways from the brain surface to the ventricles. Subcortical penetrating arteries (scop) traverse the cortex with minimal tapering to the subcortical white matter^31–33^ and to periventricular parenchyma in some cases^31^. Long branches of relatively constant diameter originating from major ventral surface vessels traverse the caudate putamen and other subcortical structures, many extending to the periventricular parenchyma^31,32^.

The presence of perivascular pathways from the exterior surface of the brain to tissue surrounding the ventricles along with prior repeated observations of rapid tracer uptake along penetrating arterioles raises the possibility of CSF transport from the subarachnoid space to the ventricles via the PVS. In this case, parenchymal waste would be at least partially cleared to the ventricles by CSF return flow, forming a brain-wide clearance circulation. Although slightly different from the glymphatic circulation proposed in Iliff, et al.^3^ in which inflowing periarterial CSF flows through the parenchyma and eventually clears via perivenular spaces, these circulations are not mutually exclusive. Unfortunately, our MRI data do not indicate the direction of contrast agent transport to directly support perivascular CSF return to the ventricles. In our experiment, it is possible that perivascular transport during the infusion proceeded from the ventricles outward due to elevated ventricular pressure as opposed to traveling along the ventricular network to the subarachnoid space before uptake into the PVS. However, these experiments were conducted with the goal of outlining the perivascular network, not to track the natural evolution of tracer over time. The former required the infusion of a large amount of contrast agent to develop sufficient contrast in the extensive perivascular network for visualization with MRI. It is nonetheless worth noting that a previous infusion study using a higher flow rate (1.6 μL/min) for a longer period (60 min) produced only subtle changes in intracranial pressure^34^. The combination of in vivo MRI during contrast agent infusion into CSF and ex vivo whole-brain imaging at higher resolution is needed to observe brain-wide perivascular circulation and evaluate the hypothesized flow patterns described above.

### Parenchymal waste clearance

The glymphatic theory posits that waste is cleared from the parenchyma by bulk flow of interstitial fluid from PVS surrounding arteries to PVS surrounding veins^2^. This assumption of flow is seemingly at odds with evidence from real-time iontophoresis (RTI) studies indicating that diffusion is the primary mode of solute transport in parenchyma^35^ and was soon challenged by computational studies suggesting unphysical pressure gradients would be required to drive advective parenchymal transport^6,36–38^. A later computational modeling study demonstrated how slow interstitial flow could explain errors observed in earlier RTI studies and argued that parenchymal transport is likely a combination of advection and diffusion^39^.

In this study, we consider the matter of parenchymal clearance from a geometric standpoint made possible by our perivascular space segmentation. By calculating the shortest distance from every parenchyma voxel to either the brain surface or the ventricles, the spatial distribution of minimum clearance distance (MCD) from parenchyma to the nearest CSF space was determined. For Rat 4, the MCD was on average 0.784 mm corresponding to a diffusion time scale for Aβ ($D^{*}$ = 62.3 μm^2^/s^28^) of 2.74 h. By considering the PVS an extension of the CSF compartment, the average MCD was reduced to 0.169 mm and the diffusion time scale for Aβ was reduced over 21-fold to 7.67 min. The pervasive nature of the perivascular network and its rapid, primarily advective, solute exchange with the CSF appears to render diffusion a viable mechanism of Aβ clearance from parenchyma. This possibility is analogous to the systemic diffusive exchange of dissolved gases between the bloodstream and tissue and has been raised in the context of PVS-mediated clearance in previous reviews^40,41^. Moreover, the average MCD was likely overestimated due to smaller perivascular segments requiring higher resolution to distinguish. Previously reported spatial distributions of Peclet number in the rat brain for intrathecally infused gadoteric acid (Gd-DOTA) derived from DCE-MRI images suggest a transition from mainly advective transport in CSF spaces and larger PVS to transport that is mainly diffusive in the parenchyma^42^. This is consistent with hydraulic network models of the PVS and parenchyma^38,43^ which predict mostly moderate to high Peclet numbers in PVS ($Pe$ > 0.1) but mostly low Peclet numbers ($Pe$ < 0.1) in parenchyma. Of course, the reduction in clearance distance caused by the perivascular network also decreased the clearance time scale assuming purely advective parenchymal transport, although less dramatically, by a factor of 4.63. If interstitial bulk flow were indeed present, transport would proceed as a combination of advection and diffusion as dictated by the Peclet number $Pe$ for a particular substance. For instance, an interstitial velocity of 0.367 µm/s would result in equal advective and diffusive time scales ($Pe$ = 1) for Aβ over the average MCD when considering PVS to be a CSF compartment.

### Oscillatory solute dispersion in perivascular spaces

Rapid perivascular uptake of CSF tracers appears to rely on arterial pulsations^3,44,45^, but the mechanism by which pulsations drive perivascular transport is not clear^41^. By periodically varying the perivascular volume, arterial pulsations are expected to produce oscillatory fluid motion in the PVS, a prediction which has been indirectly confirmed by the oscillatory motion of perivascular tracers^46,47^. Vascular pulsations may generate net fluid motion through a peristaltic mechanism^48^ although this notion has been challenged by computational modeling results^6,49^ and theoretically by noting the pulse wavelength is much longer than the typical perivascular segment length^38^. Oscillatory fluid motion has the potential to enhance perivascular tracer transport without net fluid flow through Taylor dispersion^50^ which can be approximated as an increase in the effective diffusivity of the tracer. Dispersive transport in PVS has been addressed in recent computational studies^6,7,51^. Asgari, et al.^6^ predicted an increase in effective diffusivity greater than 27% while Troyetsky, et al.^7^ predicted a more modest increase of about 5%.

Here, we investigated dispersive perivascular uptake of albumin by considering the perivascular space a straight, semi-infinite channel adjacent to a well-mixed CSF compartment and tracking the motion of advancing tracer fronts with fractions $\alpha$ of the CSF albumin concentration. Advancing fronts with lower concentration moved more quickly than fronts with higher concentration (see Eq. (6)). For instance, the $\alpha$ = 0.1 front advanced at slightly over 10 μm/s initially and covered about 1 mm in 40 min whereas the $\alpha$ = 0.8 front advanced at just under 2 μm/s initially and covered only about 150 μm in the same amount of time (Fig. 5c). This predicted penetration distance is shorter than the lengths of many perivascular segments (2–4 mm) showing strong, relatively uniform contrast agent enhancement after the 40 min infusion (Fig. 5b). The signal in these segments did not exhibit axial variations in concentration as would be expected from a dispersive transport process. Previously observed uptake into periarterial spaces appears to proceed faster than even the lowest concentration ($\alpha$ = 0.1) front considered in the dispersive transport analysis. Microspheres move in the perivascular spaces surrounding pial arteries at 18.7 μm/s^4^ on average whereas the $\alpha$ = 0.1 front drops to 3.43 μm/s after 10 s and to 1.09 μm/s after 100 s. The front velocities in dispersive transport also depend on molecular weight, contrary to evidence of molecular weight independent transport in periarterial spaces^18^. Estimates of solute speed in superficial CSF spaces including PVS derived from DCE-MRI by optimal mass transport theory (rOMT) are slower (~ 0.2 μm/s)^42^, but these estimates are derived from voxels larger than individual PVS and likely do not reflect perivascular velocities due to volume averaging. The average Peclet number in the same CSF region was nonetheless about 80, indicating the predominance of advective transport^42^. Together these results suggest that advection, rather than dispersion, was the primary perivascular uptake mechanism for the tracer in our experiment and in previous CSF infusion studies with comparable molecular weight tracers. Careful experiments by other groups involving a double-syringe system to maintain constant CSF volume also indicate that uptake is not driven by elevated infusion pressure^52^.

Dispersion will naturally make a greater transport contribution for higher diffusivity tracers in shorter perivascular segments. For instance, the $\alpha$ = 0.5 front for Aβ monomer ($D$ = 180 μm^2^/s)^28^, being smaller than albumin, traversed 250 μm in 6.06 min ($k$ = 1.05), but required 1.62 h ($k$ = 1.05) to travel 1000 μm (Fig. 5e). The $\alpha$ = 0.5 solute front for sodium ions traversed 1000 μm in 11.36 min ($k$ = 1.05) due to oscillatory dispersion. These results suggest that oscillatory dispersion may contribute substantially to transport in short perivascular segments or the start of long perivascular segments, but it loses relevance with increasing perivascular segment length and solute molecular weight. Even for the higher dispersive enhancement ($k$ = 1.7) reported for $D$ = 10 μm^2^/s in Asgari, et al.^6^ (enhancement tends to become smaller with increasing diffusivity), the Aβ monomer required 1.00 h to travel 1000 μm (Fig. 5e). Assuming the flow velocities observed in Mestre, et al.^4^ (18.7 μm/s) are present in the penetrating PVS observed in this study, solutes would traverse 4 mm (the length of the longest perivascular segments identified) by advection alone in 3.57 min suggesting that effective CSF exchange is more feasible by solute advection in PVS.

The perivascular network visualizations and segmentations presented here provide insight into the structure and function of the glymphatic system. There are numerous long perivascular segments that extend from the ventral and dorsal surfaces of the brain to the vicinity of the ventricles and cisterns that may serve as pathways for waste clearance to these internal CSF spaces and may integrate the glymphatic circulation into the larger CSF circulation. Assuming rapid fluid exchange between the segmented PVS and CSF spaces, the PVS can effectively reduce the average minimum distance from parenchyma to the nearest CSF sink thus reducing the time scale over which parenchymal waste may diffuse that distance over 21-fold, from an estimated time scale over 2 h to one under 10 min. Furthermore, a transient solute transport analysis within the PVS suggests that oscillatory dispersion is not the predominant transport mechanism for large tracers in the longest perivascular segments observed. The assumption of rapid clearance from the PVS in the parenchymal transport analysis and the inability of dispersion to rapidly transport tracer in the long perivascular segments observed imply that flow in the PVS is required to sustain effective clearance throughout the parenchyma by diffusion. The inflowing CSF may filter into the parenchyma as has been previously posited and/or flow towards the ventricles and cisterns as suggested by the geometries of PVS observed in this study. The fluid entering the parenchyma from the PVS is expected to flow at velocities much slower than velocities observed within the pial PVS given the geometric increase in flow cross-section with distance from the penetrating PVS and the low hydraulic conductivity in parenchyma. In this scenario, parenchyma transport would proceed as a combination of advection and diffusion as dictated by the Peclet number $Pe$ for a particular substance which does not necessarily rule out diffusion as a significant, if not the predominant, transport mechanism in parenchyma.

## Limitations and future directions

The experimental design did not include a means to directly distinguish arteries and veins, making it difficult to determine whether tracer was in the PVS of veins. However, in Magdoom, et al.^16^ Evans blue dye was evident in the tissue surrounding the superior sagittal sinus suggesting the presence of tracer in venous PVS. Only a few infusion studies have shown tracer surrounding veins^1,2,17^. An in vivo MR angiography experiment prior to ventricular infusion could distinguish arteries and veins, albeit at lower resolution, and guide the interpretation of ex vivo high-resolution images of the PVS.

The maps of PVS are sufficiently resolved to analyze network connectivity, define segment centerlines, measure segment lengths, and calculate changes in MCD. However, the segmentations cannot provide an accurate readout of perivascular volume because of volume averaging within the 40-μm voxels. Contrast agent within perivascular structures smaller than 40 μm has the potential to affect the signal from multiple 40-μm voxels. The true size of the PVS is likely smaller than their apparent size in the ex vivo images because of volume averaging and contrast agent diffusion from the PVS into the parenchyma over the course of the infusion. However, albumin’s relatively large size (66 kDa) limits its diffusion in brain tissue (16.3 μm^2^/s^53^) and may hinder its passage between astrocytic endfeet lining the exterior perivascular boundary^2^. The larger apparent size of the PVS produces an underestimation of the MCD. If a 17.85 μm diameter vessel lied at a shared vertex among four adjacent voxels which are segmented as perivascular voxels because of signal enhancement due to volume averaging and radial contrast agent diffusion, the minimum in-plane clearance distance would be underestimated by at most 19.36 μm. Assuming the majority of segmented PVS were for vessels of roughly 17.85 μm diameter^54^, taking this additional clearance distance into account would increase the average diffusive transport time scale for Aβ from 7.67 min to 9.53 min. It is reasonable to expect that such a vessel would enhance these four voxels because the albumin concentration in these voxels would be at minimum 45% of the perivascular albumin concentration due to radial albumin diffusion assuming a constant perivascular albumin concentration over 40 min (the duration of the intraventricular infusion).

The brain tissue was fixed after the intraventricular infusion procedure to prevent further albumin diffusion prior to imaging. However, fixation tends to shrink brain tissue slightly, leading to parenchyma clearance distances that are somewhat biased small. Ma, et al.^55^ report an average brain shrinkage due to formalin fixation in mice of 10.6% using volumetric measurements derived from MRI images. This is equivalent to a volume increase of 11.9% from the shrunken geometry to the in vivo geometry. Because volume increases as the cube of length, the corresponding increase in MCD is 3.81%. The diffusion time scale is proportional to length squared and would increase 7.76%, from 7.67 min to 8.26 min. The error in MCD associated with the apparent size of PVS and fixative-induced brain shrinkage produces modest changes in the diffusion time scale insufficient to undermine diffusion as an effective clearance mechanism from parenchyma given the abundance of perivascular spaces.

Extracellular spaces, including PVS, collapse following animal sacrifice presumably because of extracellular fluid influx into cells and consequent cell swelling^56,57^. It is unlikely, however, that the observed PVS are an artifact of their post-mortem collapse. The collapse of the PVS could potentially squeeze the contrast agent out of the spaces, detracting from their MRI visibility, making such spaces more difficult to see, not more apparent. Alternatively, fluid influx into cells following death might draw CSF and contrast agent into the PVS as in Du, et al.^57^, improving their visibility, but this should not outline spaces that were absent in vivo.

The ex vivo approach also prevented direct observation of perivascular transport direction and rate. In vivo imaging during the infusion would enable dynamic tracer observation at the expense of image resolution. As an alternative or addition, ex vivo images could be acquired following infusions of various durations. This approach would allow indirect observation of perivascular transport direction and rate without sacrificing imaging resolution. In the oscillatory transport analysis, perivascular inflow from the SAS driven by vessel pulsations or other sources of pressure gradients were not explicitly modeled. These factors could be incorporated into a model of perivascular flow and solute transport that includes the segmented geometry of PVS to further probe the mechanics of the glymphatic system by comparing simulated solute distributions with data from the in vivo and ex vivo experiments described above.

## Methods

### Magnetic resonance imaging experiments

All animal procedures were approved by the University of Florida Institutional Animal Care and Use Committee and comply with relevant guidelines and regulations. The methods reported herein adhere to the recommendations in the ARRIVE guidelines. In Magdoom, et al.^16^, high-resolution magnetic resonance images (40 ${\upmu }$m isotropic voxels) of PVS in five excised whole rat brains were acquired. Gadolinium-labeled human serum albumin, Gd-DTPA-albumin^58^, was infused into the lateral ventricle of each anesthetized rat (4% isoflurane in 1 L/min oxygen) at a rate of 1.5 μL/min for 40 min to allow distribution into the PVS. The animal was immediately exsanguinated then sacrificed via vascular perfusion with 0.9% sodium chloride solution followed by 4% formaldehyde fixative solution to crosslink the tracer to the brain tissue. After 2.5–3 days storage at 4 °C, the rat brain was excised and placed in Fluorinert oil (FC-43, 3 M Corp., St. Paul, MN, USA) in preparation for high-resolution imaging at 17.6 T (Bruker Biospin, Billerica, MA, USA). A $T_{1}$-weighted 3D gradient echo image with a FOV of 20 mm × 16 mm × 12 mm, a matrix size of 500 × 400 × 300, TR = 100 ms, TE = 0.3 ms, flip angle = 50°, and 7 averages was acquired in approximately 24 h. Two naïve control animals underwent the same experimental and imaging protocols in all ways except the stereotaxic surgery and intraventricular infusion. All animals (n = 7) were 2-month old male Sprague–Dawley rats weighing 280 – 300 g.

**Figure 6:**
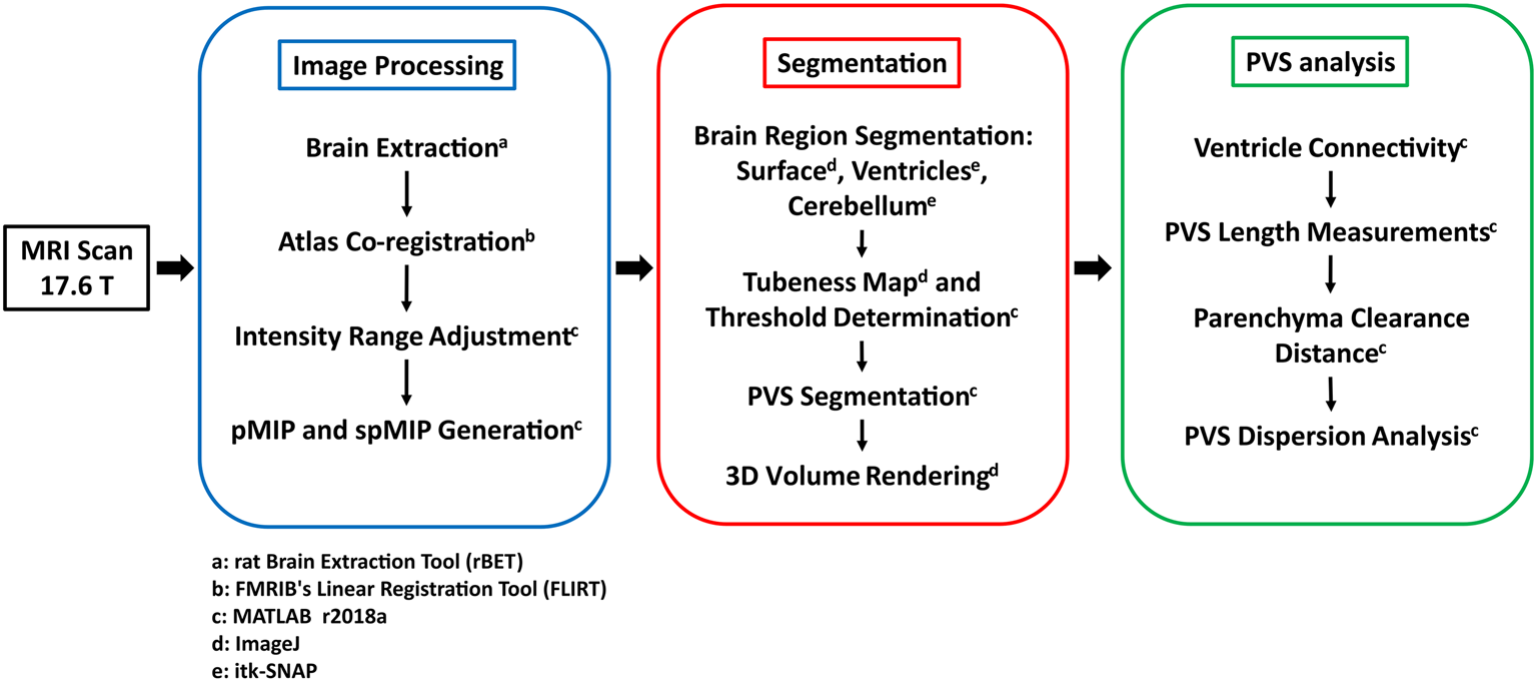
Image analysis workflow. Following the 24 h MRI session at 17.6 T, the brain was extracted and visualized with a custom maximum intensity projection technique (blue box). Then, brain regions and the PVS were segmented and rendered in 3D (red box). Lastly, PVS-ventricle connectivity and transport were analyzed (green box). The software applications used in each step, as indicated by the superscript letters, are as follows: (**a**) rodent Brain Extraction Tool (rBET); (**b**) FMRIB's Linear Registration Tool (FLIRT); (**c**) MATLAB r2018a; (**d**) ImageJ; (**e**) itk-SNAP.

The rat brain was isolated in the MR images with rodent brain extraction tool, rBET^59^, and registered to Swanson’s atlas^60^ with linear image registration tool, FLIRT^61^, before maximum intensity projections (MIPs) were produced in ImageJ^62,63^ as in Magdoom, et al.^16^. While this demonstrated the extent of perivascular uptake throughout the brain, further analysis of the perivascular network was made possible by the custom visualization and segmentation workflow presented in this study (Fig. 6). Additional details regarding the animal experiments and imaging protocol can be found in Magdoom, et al.^16^.

### Perivascular space visualization

The ventricles and PVS appear bright in the $T_{1}$-weigted 3D images because Gd-DTPA-albumin reduces $T_{1}$^16^. Despite the resulting contrast with surrounding tissue, the PVS are not readily apparent due to their small size, sometimes spanning a single voxel, and varied orientations. Computing a maximum intensity projection (MIP) of the data, as reported previously by Magdoom, et al.^16^, results in a set of projection images in which the brightest PVS and the ventricles are more apparent. However, smaller, dimmer PVS may not be projected and PVS may be occluded by the bright ventricles in the center of the brain. To overcome these limitations, partial maximum intensity projections (pMIPs) were created by projecting through 30-voxel thick rectangular volumes parallel to the coronal, sagittal, and transverse planes. Because continuous perivascular segments were often present in consecutive projections, shifting pMIPs (spMIPs) were used to visualize the full length of these structures throughout the brain (see Supplemental Video S1 to S6). In an spMIP, the projection span is shifted by a single voxel for each 30-voxel projection image instead of by 30 voxels as in a pMIP.

### Perivascular space segmentation

The PVS pose a segmentation challenge because of their small size and the surrounding tissue’s non-uniform signal strength. Because the PVS surround blood vessels, they appear like slender, tubular, bright structures in many brain regions with a range of mean intensity values. To better distinguish them from these varied surroundings, a measure of the tubular quality or tubeness of the intensity field surrounding each voxel was computed in ImageJ^64,65^. The tubeness of a given voxel was defined as the geometric mean of the two most negative eigenvalues of the Hessian matrix for that voxel. The Hessian matrix elements are the second spatial derivatives of the image intensity field^65^

1 $$H_{ij} = \frac{{\partial^{2} I}}{{\partial x_{i} \partial x_{j} }}$$

The matrix element subscripts $i$ and $j$ indicate the axes along which the differentiations are performed (see Supplementary Table S2 for all variables and parameters). The eigenvectors and eigenvalues of the Hessian matrix are thus the directions and magnitudes of the principal curvatures for the intensity field. For a region of high intensity pixels with lower intensity surroundings, the eigenvalues are negative, $0 > \lambda_{1} > \lambda_{2} > \lambda_{3}$, and each eigenvalue is the image intensity curvature along its associated eigenvector. For a tubular intensity field, $\lambda_{1} = 0$ and $\lambda_{2} = \lambda_{3} \ll 0$^65^ because the intensity does not vary along the tube axis, but has negative curvature perpendicular to this axis. Accordingly, in the ImageJ implementation, tubeness is defined as $\sqrt {\lambda_{2} \lambda_{3} }$ and increases in value for more tubular intensity fields^64^. In this implementation, the tubeness field can be made sensitive to tubular structures of a certain size by applying a Gaussian filter prior to computing tubeness. Because many PVS only span a single voxel, the standard deviation for the Gaussian filter was set to 40 μm. The tubeness calculation is a variety of Frangi filtering^26^, a technique often applied to clinical MR images for perivascular space segmentation in humans^20–22,25^.

**Figure 7:**
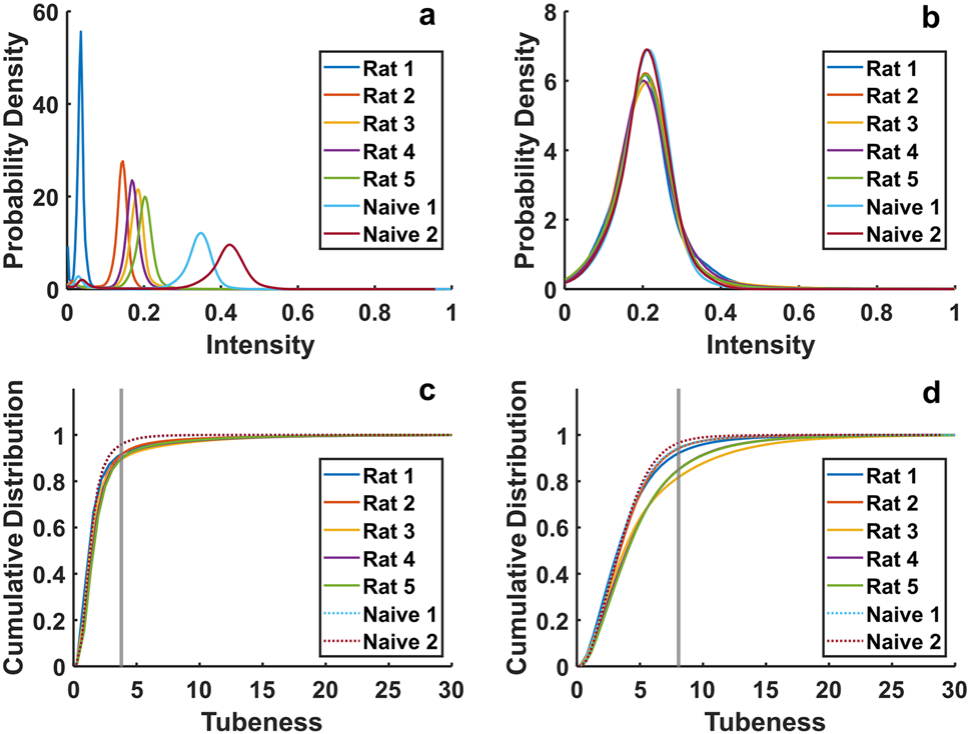
Signal intensity and tubeness distributions. (**a**) Signal intensity from the brains with contrast agent (Rat 1–5) and without contrast agent (Naïve 1–2). The signal intensity for each animal was mapped linearly between 0 and 1. (**b**) Signal intensity following intensity range remapping to align signal distribution peaks and create better perivascular contrast with the surrounding tissue. (**c–d**) Tubeness distribution for the brain (**c**) interior and (**d**) surface. The gray vertical line is the average of the tubeness values corresponding to the 0.95 cumulative distribution value in each naïve brain. This tubeness value is greater than the tubeness in 95% of the naïve voxels on average.

The perivascular space segmentations were generated by computing the tubeness of the 3D images and selecting voxels with tubeness above a threshold. Because the contrast agent greatly increased signal strength above levels in the naïve brains, the peaks of the intensity distribution for each brain were aligned by adjusting the data range before determining the tubeness threshold (Fig. 7a, b).

The threshold was set to exceed the tubeness values of 95% of voxels in the naïve brains (tubeness = 3.75; Fig. 7c). This was done to exclude background tubeness not associated with the uptake of contrast agent in the PVS. The brain’s exterior surfaces had higher background tubeness levels because the interface between the parenchyma and the dark surroundings produced high spatial intensity gradients in the naïve brains. Therefore, a higher tubeness threshold (tubeness = 8.08) was used to segment the PVS of surface blood vessels (Fig. 7d).

**Figure 8:**
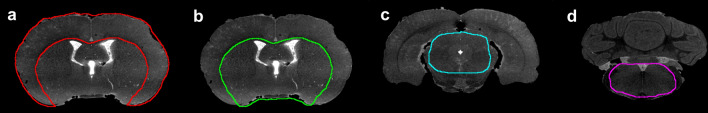
Brain volumes of interest (VOI) for perivascular space segmentation analysis. A coronal slice from the VOI in the (**a**) cortex, (**b**) striatum, (**c**) periaqueductal tissue, and (**d**) brain stem. Each VOI spans 30 coronal slices.

Several segmentations of anatomical structures including the ventricles and cerebellum were created in itk-SNAP^66^ using both manual outlining and the “snake” semi-autosegmentation tool. The ventricle network appeared bright because of the infused contrast agent which facilitated segmentation by comparison with the Paxinos rat brain atlas^27^. The tubeness-based segmentation of the PVS was improved by removing the cerebellum because its internal boundaries made PVS difficult to distinguish by means of tubeness alone. Volumes of interest (VOI) in the cortex, striatum, periaqueductal tissue, and the brain stem spanning 30 coronal slices each were manually outlined to compare perivascular voxel volume percent and mean MCD in these anatomical regions across animals (Fig. 8). The cortex and striatum VOI occupied the same set of 30 slices, and the striatum was defined as the tissue ventral and medial to the cortex within these slices.

### Ventricle connectivity and perivascular segment length

The connections between the PVS and ventricle network were examined with a custom region-grow algorithm that iteratively expanded the ventricle segmentation into the perivascular segmentation. In each iteration, perivascular voxels immediately adjacent to a ventricle voxel were added to the growing ventricle segmentation for a total of 100 iterations. Prior to beginning this procedure, the ventricle segmentation was enlarged by three voxels to capture PVS within 208 µm of the ventricular surface, the diagonal distance across three 40-μm cubical voxels aligned diagonally. A margin of three voxels was selected because this length is short enough to enable relatively rapid transport of perivascular tracers into the ventricles. This produced a segmentation that included the ventricles and portions of the perivascular network continuous with perivascular segments near the ventricles.

PVS length estimates were made for a selection of long, continuous segments penetrating from the ventral and dorsal brain surfaces using a modified region-grow algorithm. A seed voxel was placed at the surface end of each perivascular segment and the centroid of voxels added after each growth iteration was recorded. The distances between these centroids were computed and summed to yield an estimate of the total perivascular segment length.

### Perivascular and parenchymal transport analyses

The extent to which the perivascular network facilitates exchange between CSF and interstitial fluid was quantified by calculating the distance between parenchyma voxels and the nearest CSF compartment, $L$, both considering and not considering the PVS to be a CSF space. In this analysis, the PVS were considered CSF compartments given numerous observations of rapid exchange between PVS and larger CSF spaces. The minimum distances were determined by computing a Euclidean distance transform^67^ of the CSF segmentation in MATLAB (MATLAB v. 9.4.0.813654 (R2018a), The MathWorks, Inc., Natick, MA). Clearance time scales $\tau_{d}$ and $\tau_{a}$ for a range of relevant solute diffusivities were computed assuming either purely diffusive (Eq. (2a)) or advective (Eq. (2b)) transport through the parenchyma.

2a $$\tau_{d} = L^{2} /D^{*}$$

2b $$\tau_{a} = L/u$$

In Eq. (2a), $D^{*}$ is the effective diffusivity of the solute in parenchyma and $u$ is the interstitial velocity magnitude. $D^{*}$ is lower than the solute free diffusivity $D$ because of parenchymal structures that hinder random solute motion. The reduction in parenchymal clearance time due to the perivascular network was estimated for a physiologically relevant range of $D^{*}$ values (10^1^–10^3^ µm^2^/s^37^) including that of Aβ (62.3 μm^2^/s^28^).

A perivascular transport model was developed to investigate oscillatory solute dispersion in the longest perivascular segments observed in this study. This model assumes the CSF in the subarachnoid space is well mixed and maintains a constant solute concentration $C_{1}$ while the perivascular segments contain free fluid initially at concentration $C_{0}$ = 0. Because the perivascular segments are long and slender, a one-dimensional semi-infinite geometry (Fig. 5a) was considered for which the origin is located at the interface between the CSF reservoir and the perivascular segment. Dynamic changes in perivascular geometry due to vessel wall motion were not modeled since these are small relative to the perivascular gap width^3,68^. Dispersion due to oscillatory flow in an annulus has been previously modeled as an enhanced diffusion process where the solute free diffusivity $D$ is increased by a factor $k$^6,7,50^. Perivascular inflow from the SAS driven by vessel pulsations or other sources of pressure gradients were not explicitly modeled; only the dispersive effect of oscillatory flow on the evolution of solute concentration was considered. Therefore, the governing equation for this scenario is

3 $$\frac{\partial C}{{\partial t}} = kD\frac{{\partial^{2} C}}{{\partial x^{2} }}$$

Given the absence of a geometric length scale, a self-similar solution was sought for non-dimensional concentration $\alpha$ in terms of variable $\eta$ where

4a $$\alpha = \frac{{C - C_{0} }}{{C_{1} - C_{0} }}$$

4b $$\eta = \frac{x}{{\sqrt {4kDt} }}$$

The self-similar solution is defined in terms of the error function as

5a $$\alpha = 1 - {\text{erf}}\left( \eta \right)$$

5b $${\text{erf}}\left( \eta \right) = \frac{2}{\sqrt \pi }\mathop \smallint \limits_{0}^{\eta } e^{{ - z^{2} }} dz$$

The position $x$ and velocity $v$ of the material point with a fraction $\alpha$ of the CSF concentration $C_{1}$ in the perivascular segment over time is given by

6a $$x = 2\sqrt {kD} {\text{erf}}^{ - 1} \left( {1 - \alpha } \right)\sqrt t$$

6b $$v = \sqrt {kD} {\text{erf}}^{ - 1} \left( {1 - \alpha } \right)\sqrt {1/t}$$

Equation (6) represents the kinematics of dispersive tracer uptake into the PVS. Bovine serum albumin ($D$ = 83 μm^2^/s^53^) with maximum enhancement $k$ = 1.05^7^ and $k$ = 1.7^6^ was considered a representative large molecular weight tracer.

## Supplementary Information

Supplementary Information 1.

Supplementary Information 2.

Supplementary Information 3.

Supplementary Information 4.

Supplementary Information 5.

Supplementary Information 6.

Supplementary Information 7.

Supplementary Information 8.

## Data Availability

The data is available upon request from the corresponding author.
