# Supplementary figures and images for: Perivascular network segmentations derived from high-field MRI and their implications for perivascular and parenchymal mass transport in the rat brain
Source: Sci Rep. 2023 Jun 6;13:9205. doi: 10.1038/s41598-023-34850-0 (PMC10244386; doi:10.1038/s41598-023-34850-0)

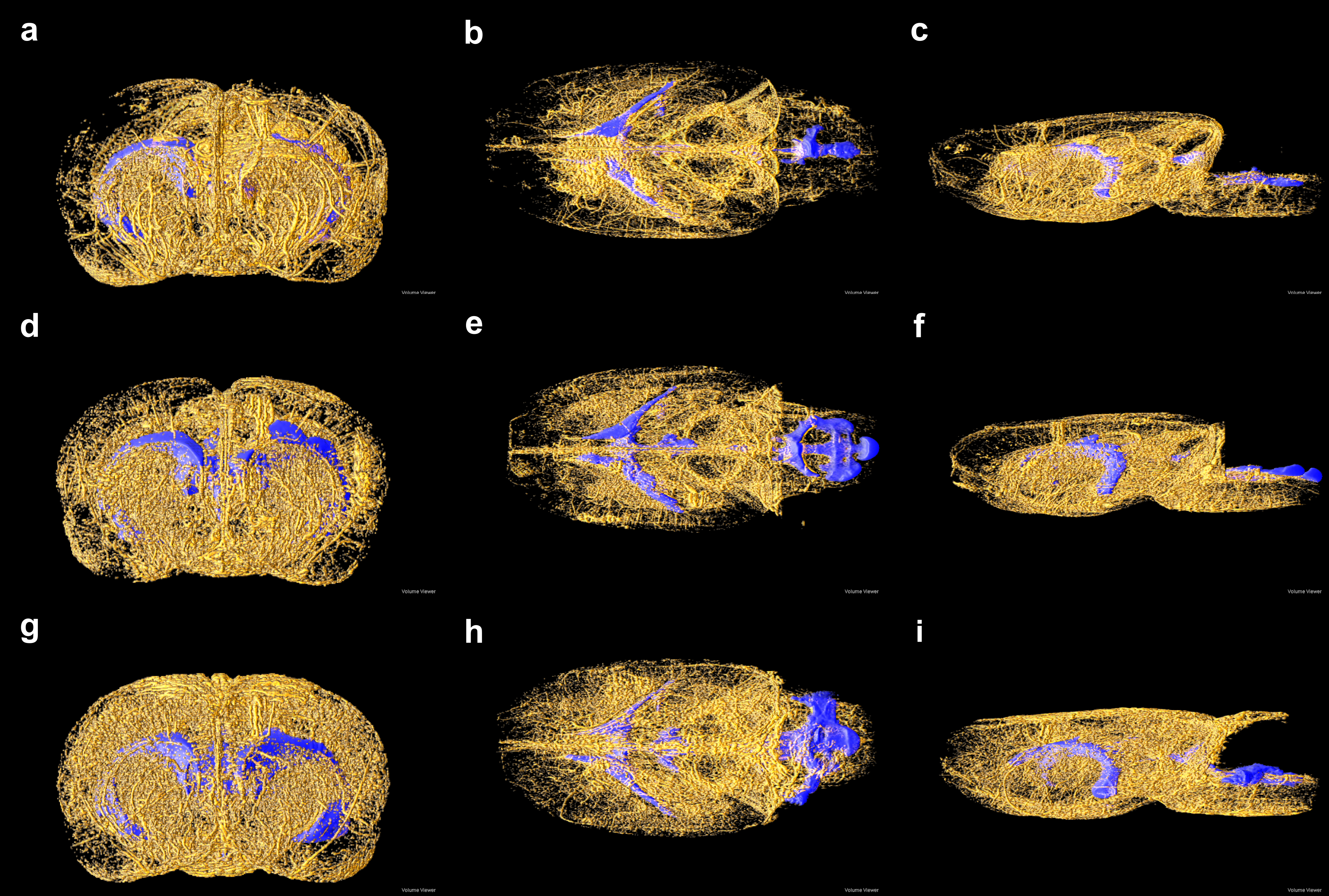

Supplement: Supplementary file 1 — Supplementary Information 1. [file 41598_2023_34850_MOESM1_ESM.png]

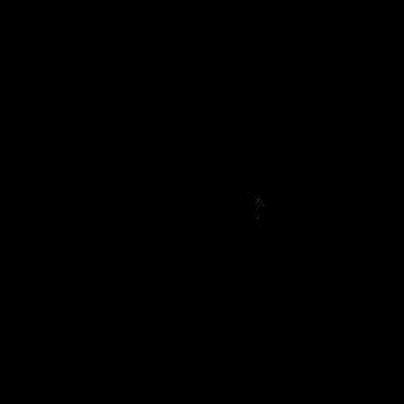

Supplement: Supplementary file 3 — Supplementary Information 3. [file 41598_2023_34850_MOESM3_ESM.gif]

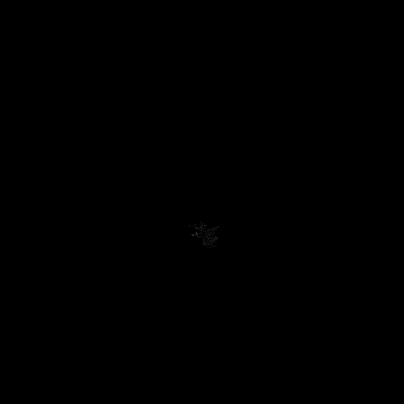

Supplement: Supplementary file 4 — Supplementary Information 4. [file 41598_2023_34850_MOESM4_ESM.gif]

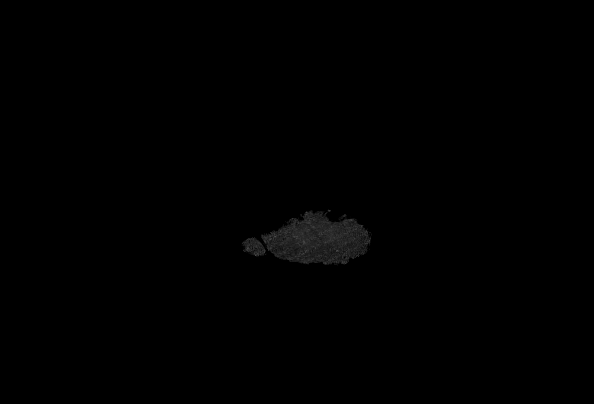

Supplement: Supplementary file 5 — Supplementary Information 5. [file 41598_2023_34850_MOESM5_ESM.gif]

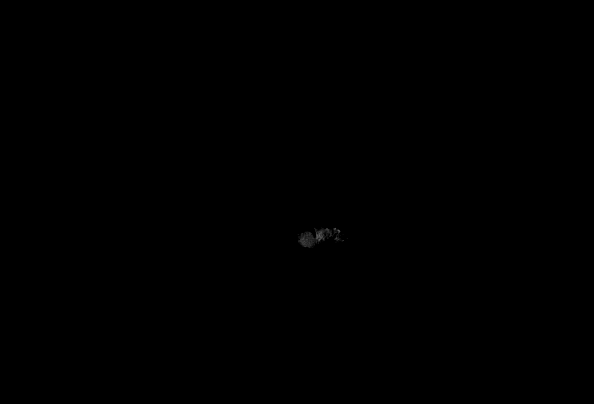

Supplement: Supplementary file 6 — Supplementary Information 6. [file 41598_2023_34850_MOESM6_ESM.gif]

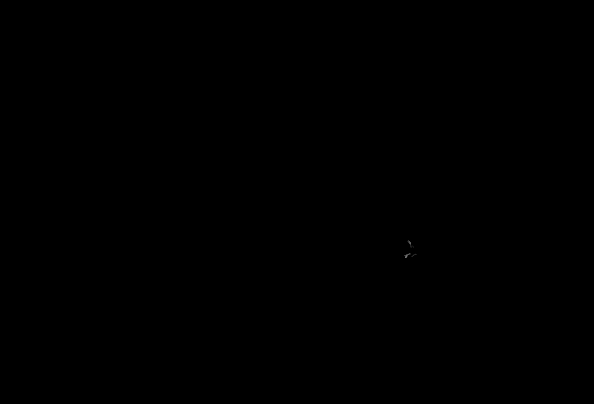

Supplement: Supplementary file 7 — Supplementary Information 7. [file 41598_2023_34850_MOESM7_ESM.gif]

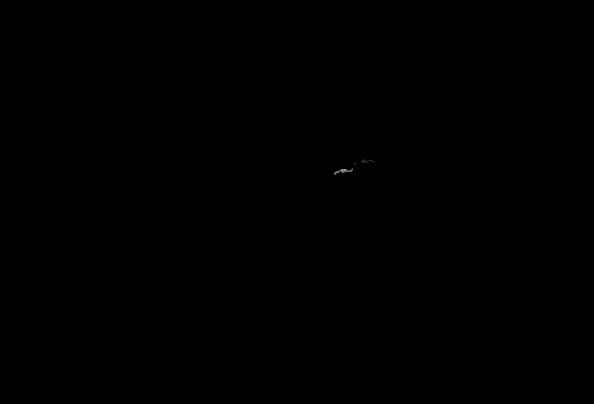

Supplement: Supplementary file 8 — Supplementary Information 8. [file 41598_2023_34850_MOESM8_ESM.gif]
