# Supplementary material for: Perivascular network segmentations derived from high-field MRI and their implications for perivascular and parenchymal mass transport in the rat brain
Source: Sci Rep. 2023 Jun 6;13:9205. doi: 10.1038/s41598-023-34850-0 (PMC10244386; doi:10.1038/s41598-023-34850-0)
Supplement: Supplementary file 2 — Supplementary Information 2. [file 41598_2023_34850_MOESM2_ESM.pdf]

Supplementary Information

Perivascular network segmentations derived from high-field MRI and their implications for perivascular and parenchymal mass transport in the rat brain

Julian A. Rey[1], Uzair M. Farid[1], Christopher M. Najjoum[1], Alec Brown[2], Kulam Najmudeen Magdoom[1], Thomas H. Mareci[2], Malisa Sarntinoranont[1]*

[1]Department of Mechanical and Aerospace Engineering, University of Florida, Gainesville, Florida, USA.

[2]Department of Biochemistry and Molecular Biology, University of Florida, Gainesville, Florida, USA.

Supplementary Table S1. List of abbreviations

Abbreviation	Full name
Aβ	amyloid-beta
ac	ambient cistern
acer	anterior cerebral artery
ach	anterior choroidal artery
aica	inferior anterior cerebellar artery
astr	anterior striate arteries
azac	azygos anterior cerebral artery
azp	azygos pericallosal artery
bas	basilar artery
cop	cortical penetrating arteries
crhv	caudal rhinal vein
cbv	cisternal blood vessels
CSF	cerebrospinal fluid
dpaq	dorsal periaqueductal arteries
ictd	internal carotid artery
lhia	longitudinal hippocampal artery
lpaq	lateral periaqueductal arteries
lv	lateral ventricles
mcer	middle cerebral artery
MCD	minimum clearance distance
mmd	median medullary arteries
mmes	median mesencephalic arteries
mpn	median pontine arteries
MR	magnetic resonance
MRI	magnetic resonance imaging
mstr	medial striate arteries
nt	needle track
pcer	posterior cerebral artery
pMIP	partial maximum intensity projection
pstr	posterior striate arteries
pva	pontine ventral arteries
PVS	perivascular spaces
rha	rhinal artery
RTI	real-time iontophoresis
scba	superior cerebellar artery
scol	superior colliculus
scop	subcortical penetrating arteries
spMIP	shifting partial maximum intensity projection
str	striate arteries
thp	thalamo-perforating arteries
trhi	trans-hippocampal arteries
vth	ventral thalamic arteries
3v	third ventricle
4v	fourth ventricle

Supplementary Table S2. List of variables and parameters

Symbol	Description	Value
H	Hessian matrix	
I	Image intensity	
x_i	Image coordinate axes	
λ_i	Hessian matrix eigenvalue	
τ_d	Diffusive clearance time scale	
τ_d	Advective clearance time scale	
L	Average minimum clearance distance	
D^*	Effective solute diffusivity in parenchyma	Aβ: 6.23 x 10^1 μm^2/s (Waters [1])<br>Range: 10^1 - 10^3 μm^2/s (Holter, et al. [2])
u	Interstitial fluid velocity	
C	Perivascular solute concentration	
t	Time elapsed for perivascular transport	
x	Position along perivascular segment	
ν	Velocity of advancing solute front	
D	Free solute diffusivity in perivascular space	Bovine Serum Albumin: 8.3 x 10^1 μm^2/s (Tao and Nicholson [3])
k	Dispersive transport enhancement	1.05 (Troyetsky, et al. [4]); 1.7 (Asgari, et al. [5])
α	Non-dimensional perivascular solute concentration	
C_1	Cerebrospinal fluid solute concentration	
C_0	Initial perivascular solute concentration	
η	Self-similar parameter	

Supplementary Figure S1. Perivascular and ventricle segmentations for the remaining animals. All PVS (gold) and the ventricles (blue) are rendered in 3D for Rat 1 (a-c), Rat 2 (d-f), and Rat 5 (g-i). Panels (a), (d), and (g) are a rostral view of the coronal plane, panels (b), (e), and (h) are a dorsal view of the transverse plane, and panels (c), (f), and (i) are a left view of the sagittal plane.

Supplementary Video S1. Coronal shifting partial maximum intensity projections (spMIPs) for Rat 3. Each frame is a 30-slice maximum intensity projection along the anteroposterior axis, and the 30-slice range is shifted by a single voxel from frame to frame. The animation is played at 10 fps.

Supplementary Video S2. Coronal shifting partial maximum intensity projections (spMIPs) for Rat 4. Each frame is a 30-slice maximum intensity projection along the anteroposterior axis and the 30-slice range is shifted by a single voxel from frame to frame. The animation is played at 10 fps.

Supplementary Video S3. Sagittal shifting partial maximum intensity projections (spMIPs) for Rat 3. Each frame is a 30-slice maximum intensity projection along the mediolateral axis and the 30-slice range is shifted by a single voxel from frame to frame. The animation is played at 10 fps.

Supplementary Video S4. Sagittal shifting partial maximum intensity projections (spMIPs) for Rat 4. Each frame is a 30-slice maximum intensity projection along the mediolateral axis and the 30-slice range is shifted by a single voxel from frame to frame. The animation is played at 10 fps.

Supplementary Video S5. Transverse shifting partial maximum intensity projections (spMIPs) for Rat 3. Each frame is a 30-slice maximum intensity projection along the dorsoventral axis, and the 30-slice range is shifted by a single voxel from frame to frame. The animation is played at 10 fps.

Supplementary Video S6. Transverse shifting partial maximum intensity projections (spMIPs) for Rat 4. Each frame is a 30-slice maximum intensity projection along the dorsoventral axis and the 30-slice range is shifted by a single voxel from frame to frame. The animation is played at 10 fps.

References

[1] J. Waters, "The concentration of soluble extracellular amyloid-beta protein in acute brain slices from crnd8 mice," *Plos One,* vol. 5, p. 16, Dec 2010.

[2] K. E. Holter, B. Kehlet, A. Devor, T. J. Sejnowski, A. M. Dale, S. W. Omholt*, et al.*, "Interstitial solute transport in 3d reconstructed neuropil occurs by diffusion rather than bulk flow," *Proceedings of the National Academy of Sciences of the United States of America,* vol. 114, pp. 9894-9899, Sep 2017.

[3] L. Tao and C. Nicholson, "Diffusion of albumins in rat cortical slices and relevance to volume transmission," *Neuroscience,* vol. 75, pp. 839-847, Dec 1996.

[4] D. E. Troyetsky, J. Tithof, J. H. Thomas, and D. H. Kelley, "Dispersion as a waste-clearance mechanism in flow through penetrating perivascular spaces in the brain," *Scientific Reports,* vol. 11, p. 12, Feb 2021.

[5] M. Asgari, D. de Zelicourt, and V. Kurtcuoglu, "Glymphatic solute transport does not require bulk flow," *Scientific Reports,* vol. 6, p. 11, Dec 2016.
